# Validation of experimental and gradient boosting regressor model for predicting performance, combustion, emission, and biomedical implications of cerium oxide (CeO₂)-augmented B20 biodiesel blends derived from used temple oil

**DOI:** 10.1186/s13065-025-01693-x

**Published:** 2025-12-05

**Authors:** Ganesh Krishnappa, Devarahalli Kempegowda Ramesha, Seetharamapura Balaji Anjappa, Praveena Bindiganavile Anand, Abdulfatah Abdu Yusuf, Muhammad Imam Ammarullah

**Affiliations:** 1 Department of Mechanical Engineering, Nitte Meenakshi Institute of Technology, NITTE (Deemed to be University), Bangalore, 560064 Karnataka India; 2 Department of Mechanical Engineering, University of Visvesvaraya College of Engineering, Bengaluru, 560001 Karnataka India; 3 Department of Mechanical Engineering, Brindavan College of Engineering, Bangalore, 562175 Karnataka India; 4 Department of Mechanical Engineering, College of Engineering, University of Liberia, Monrovia, 1000 Montserrado, Liberia; 5 Bioengineering and Environmental Sustainability Research Centre, University of Liberia, Monrovia 1000 Montserrado, Liberia; 6 Department of Mechanical Engineering, Faculty of Engineering, Universitas Diponegoro, Semarang, 50275 Central Java Indonesia

**Keywords:** Biodiesel, Nano additives, Gradient boosting regressor model, Biomedical

## Abstract

The global decline in fossil fuel availability and rising environmental concerns have intensified the search for sustainable alternative fuels, with biodiesel emerging as a promising option. This study investigates the performance, combustion, and emission characteristics of a B20 biodiesel blend derived from Used Temple Oil Methyl Ester (UTOME) enhanced with cerium oxide (CeO₂) nano additives. Conducted at constant speed and varying engine loads, the experiments show that CeO₂ additives significantly enhance brake thermal efficiency (BTE), with the B20UTOME100CeO₂ blend achieving efficiency levels comparable to pure diesel. Specific fuel consumption (SFC) decreases as CeO₂ concentration increases, reflecting enhanced fuel efficiency, while higher cylinder pressure (CP) and net heat release (NER) signify improved combustion processes. Emission analysis reveals substantial reductions in hydrocarbons (HC), carbon monoxide (CO), and nitrogen oxides (NO_x_), with the B20UTOME100CeO₂ blend demonstrating the lowest HC and NO_x_ emissions due to better fuel atomization, improved combustion efficiency, and lower peak combustion temperatures enabled by the CeO₂ nanoparticles. The B20UTOME100CeO₂ blend demonstrated enhanced performance at maximum load, exhibiting a 1.57% increase in Brake Thermal Efficiency, a 6.67% reduction in Specific Fuel Consumption, a 2.83% elevation in cylinder pressure, and a 5.95% greater heat release when compared to conventional diesel. Emissions analysis revealed a significant reduction in carbon monoxide by 66.67%, hydrocarbons by 13.51%, and nitrogen oxides by 0.20% relative to diesel. A gradient boosting regressor model was trained on experimental data comprising fish oil biodiesel blends with nanoparticle additives, utilizing Python libraries. The accuracy of the model is supported by a mean squared error of 0.7647 for HC emissions and R² scores of 0.9247 and 0.9882 for HC and NO_x_ emissions, respectively. Overall, the results indicate that B20UTOME with 100 ppm CeO₂ additives offers a viable alternative to diesel, providing improved thermal efficiency, enhanced combustion, and reduced harmful emissions. The marked reduction in toxic emissions, including CO, HC, and NO_x_, carries significant biomedical relevance. These pollutants are known to induce respiratory, cardiovascular, and neurological disorders upon prolonged exposure. Therefore, implementing CeO₂-enhanced biodiesel blends offers a potential strategy for protecting public health by diminishing harmful exhaust emissions.

## Introduction

The global energy demand is rapidly escalating, with projections indicating a nearly 60% increase by 2035. This surge in energy consumption has led to the depletion of fossil fuel reserves, which, if the current trend continues, are estimated to be exhausted within approximately 40 years for oil, 55 years for natural gas, and 130 years for coal [1]. Furthermore, the rising per capita fuel consumption has driven up fuel prices. Concurrently, the extensive use of fossil fuels is widely recognized as a significant contributor to environmental pollution, with the combustion process releasing harmful exhaust gases [2]. Extensive academic research has been undertaken over the previous two decades regarding the utilization of biofuel for internal combustion engines. It is estimated that there is an approximately 1.1% annual increase in energy consumption in the transportation sector globally [3]. Biofuels are alternative fuels typically derived from biomass [4], with feedstocks including crops [5], forest residues [6], and municipal waste [7]. Biofuels being biodegradable, renewable and non-toxic have emerged as an environmentally friendly alternative to conventional diesel [8]. Biodiesel, produced from edible and non-edible oils such as Calophyllum and Jatropha, has been recognized as a renewable fuel substitute offering superior combustion characteristics and a reduction in harmful emissions [9]. The direct use of vegetable oils in diesel engines is restricted by several factors, including their elevated viscosity, poor atomization efficiency, and the formation of carbon deposits that can obstruct injectors. Transesterification serves as an effective method to overcome these drawbacks by reducing viscosity and enhancing fuel production. The resultant biodiesel possesses physicochemical properties similar to diesel, making it suitable for use without requiring engine modifications [10]. The use of biodiesel results in reduced emissions of carbon dioxide, hydrocarbons (HC), and particulate matter due to its reduced sulphur levels, diminished aromatic content, and the existence of oxygen-based compounds. The increased oxygen concentration found in biodiesel promotes a more complete combustion, which in turn leads to higher combustion temperatures and an increase in the generation of nitrogen oxides [11]. Furthermore, biodiesel demonstrates improved ignition characteristics in engines compared to conventional diesel fuel, attributable to its elevated cetane number [12].

The combustion of fossil fuels in vehicles releases numerous detrimental pollutants that pose significant biomedical risks and adversely affect human health. The combustion of diesel fuel releases harmful emissions, notably soot particulates and nitrogen oxides, which contribute to environmental degradation and adverse health effects [13]. Carbon monoxide (CO) would contribute to cardiovascular problems [14], an increased risk of stroke [15], and diminished cognitive abilities [16]. Vehicle exhaust’s carbon dioxide (CO_2_) contributes to systemic inflammation [17], decreased mental acuity [18], and impaired organ function [19]. Fine particulate matter (PM_2.5_), upon deep inhalation into the lungs, is associated with chronic respiratory ailments [20], cardiovascular diseases [21], and cancer development [22]. Nitrogen dioxide (NO_2_) from exhaust gases causes damage to lung tissue [23] and compromises respiratory function [24], while polycyclic aromatic hydrocarbons (PAH) negatively impact both the respiratory [25] and nervous systems [26], also it classified as a carcinogen [27]. Persistent exposure to these vehicular emissions presents a substantial biomedical and public health challenge, highlighting the urgent need for the implementation of cleaner transportation solutions [28]. The utilization of alternative fuels like biodiesel and bioalcohol blends has demonstrated a considerable reduction in carbon monoxide, hydrocarbon, and particulate matter emissions when compared to traditional diesel [13].

The integration of nanoparticles into fuel systems is emerging as a promising strategy to significantly enhance fuel properties, leading to both reduced exhaust emissions and improved engine performance [29]. Recent studies indicate that nanoparticles can improve diesel engine fuels by reducing ignition delay [30], lowering the auto-ignition temperature [31], and shortening evaporation time [32]. The nano additives used in diesel–biodiesel blends include iron oxide (Fe₂O₃), aluminum oxide (Al₂O₃), cerium oxide (CeO₂), copper oxide (CuO), silver oxide (Ag₂O), and nonmetal oxides such as carbon nanotubes (CNTs), titanium oxide (TiO₂), and zinc oxide (ZnO) [33].Small quantities of additives such as aluminium oxide (Al₂O₃), cerium oxide (CeO₂), and carbon nanotube (CNT) also enhance fuel dispersion [34], prevent injector clogging [35], and strengthen the bonding in biodiesel-water or biodiesel-diesel emulsions [36]. The inclusion of metal oxide nanoparticles, such as Al₂O₃ and Ag₂O, in diesel and biodiesel mixtures improves combustion efficiency. This enhancement is attributed to their exceptional catalytic properties and superior thermal conductivity. Additionally, their large surface area and uniform dispersion facilitate improved fuel atomization, accelerated oxidative reactions, and a reduction in ignition delay, thereby contributing to more complete combustion [37]. Additionally, nano-additives have emerged as a promising method for controlling nitrogen oxides (NO_x_), CO, and HC emissions in biofuel-powered diesel engines [38]. The incorporation of nanoparticles such as CeO₂, Al₂O₃, titanium dioxide (TiO₂), and graphene oxide (GO) into biodiesel mixtures has been demonstrated to substantially decrease emissions of CO, HC, and NO_x_, all of which are implicated in significant biomedical issues, including respiratory [39], cardiovascular [40], and neurological ailments [41]. Enhanced combustion efficiency, facilitated by the catalytic and oxidative capabilities of these nanoparticles, promotes more thorough fuel oxidation and consequently diminishes the production of deleterious gases. For example, studies by Mofijur et al. [42] indicate that Al₂O₃ and CeO₂ nanoparticles can reduce CO emissions by as much as 60%, while graphene-based additives have achieved reductions in HC emissions surpassing 50%. These reductions in emissions are particularly advantageous in densely populated urban [43] and industrial areas [44]. Thus, biodiesel enhanced with nanoparticles not only enhances engine performance but also plays a crucial role in safeguarding public health, thereby presenting its biomedical significance.

The incorporation of heightened carbon nanotube concentrations within biodiesel compositions correlates with a notable reduction in NO_2_ emissions, potentially resulting from optimized combustion dynamics and augmented radical scavenging capabilities [45]. CeO₂ nanoparticles exhibit distinctive characteristics, including robust thermal stability [46], capacity for UV absorption [47], electrical conductivity [48], significant hardness [49], particular chemical reactivity [50], and superior oxygen retention [51]. When incorporated into a blend of corn oil methyl ester and diesel at concentrations of 25, 50, and 75 parts per million, these nanoparticles notably enhanced the fuel’s physicochemical properties [52]. The addition of CeO₂ nanoparticles to biodiesel blends leads to an enhancement of brake thermal efficiency (BTE) and a simultaneous reduction in emissions like unburned HC, CO, and NO_x_, as well as a decrease in brake specific fuel consumption (SFC) [53]. In addition to biodiesel and nanoparticle-based fuels, HC derived from pyrolysis is also gaining traction as a viable alternative [54]. Low-molecular-weight HC produced from plastics like polypropylene and polystyrene can function as fundamental components in pharmaceutical synthesis [55]. Furthermore, refined fractions from low-density polyethylene and high-density polyethylene demonstrate potential for applications in medical-grade polymers [56], coatings [57], and drug delivery mechanisms [58]. Select derivatives have also exhibited antimicrobial characteristics, highlighting their importance in the biomedical field [59].

Within the domains of engineering and scientific research, machine learning represents data-processing methodologies employed to address a diverse array of challenges, particularly in contexts where traditional modelling approaches have proven inadequate. These techniques leverage algorithms capable of extracting meaningful insights from data and generating forecasts based on that information [60]. It has been empirically demonstrated that machine learning approaches are effective in predicting the behaviour of complex systems, including engines [61]. Regression analysis is a commonly employed statistical approach for evaluating the relationship between a dependent variable and one or more independent variables. This analytical method can be applied to both linear and nonlinear problems [62]. The study utilizes a variety of machine learning techniques, including random forest regression [63], decision tree regression [64], linear regression [65], and gradient boosting [66], to generate predictive outputs. Notably, the random forest model employs a bagging approach [67], while the other three methods are based on boosting principles [68, 69]. A comparative evaluation of the algorithms is conducted, leveraging assessment metrics such as coefficient of determination, root mean squared error, and mean absolute error, to thoroughly evaluate their predictive capabilities [70]. Gradient boosting regression is a boosting algorithm that constructs models incrementally through gradient descent, aiming to minimize a differentiable loss function. It is comprised of three key elements: a loss function, a weak learner, and an additive model that combines learners to diminish error [71]. Gradient boosting is a powerful ensemble machine learning methodology, widely employed for both classification and regression tasks [72]. The gradient boosting regression technique represents a highly effective approach for modelling and forecasting, and has been extensively utilized across a broad range of domains [73]. Machine learning models, such as XGBoost, enable accurate prediction and validation of results by considering biodiesel blend ratios, nanoparticle concentrations, and engine load. These validation-based, data-driven approaches enhance result reliability and optimize fuel formulations for improved efficiency and reduced emissions [74]. Machine learning algorithms, including Random Forest and AdaBoost, have demonstrated a robust capability for validating experimental results and precisely predicting engine performance characteristics, offering enhanced reliability compared to traditional analytical methods [75]. Existing research has explored biodiesel production from waste cooking oil and other oil sources, as well as the incorporation of nanoparticles such as Al₂O₃, TiO₂, and CeO₂ to enhance combustion and reduce emissions. However, the suitability of used temple oil (UTO) as a biodiesel feedstock remains relatively unexplored, despite its unique composition and sustainable supply potential. While CeO₂ nanoparticles have been applied in diesel and conventional biodiesel, their integration with UTO-based blends is scarcely documented. Furthermore, much biodiesel research relies on traditional regression or neural network models, with limited investigation into advanced ensemble techniques like Gradient Boosting. This study aims to address these gaps by experimentally evaluating CeO₂-enhanced B20 UTO biodiesel and utilizing gradient boosting regressor modelling to predict performance, combustion dynamics, and emission characteristics.

## Materials and methodology

### Biodiesel preparation

**Fig. 1:**
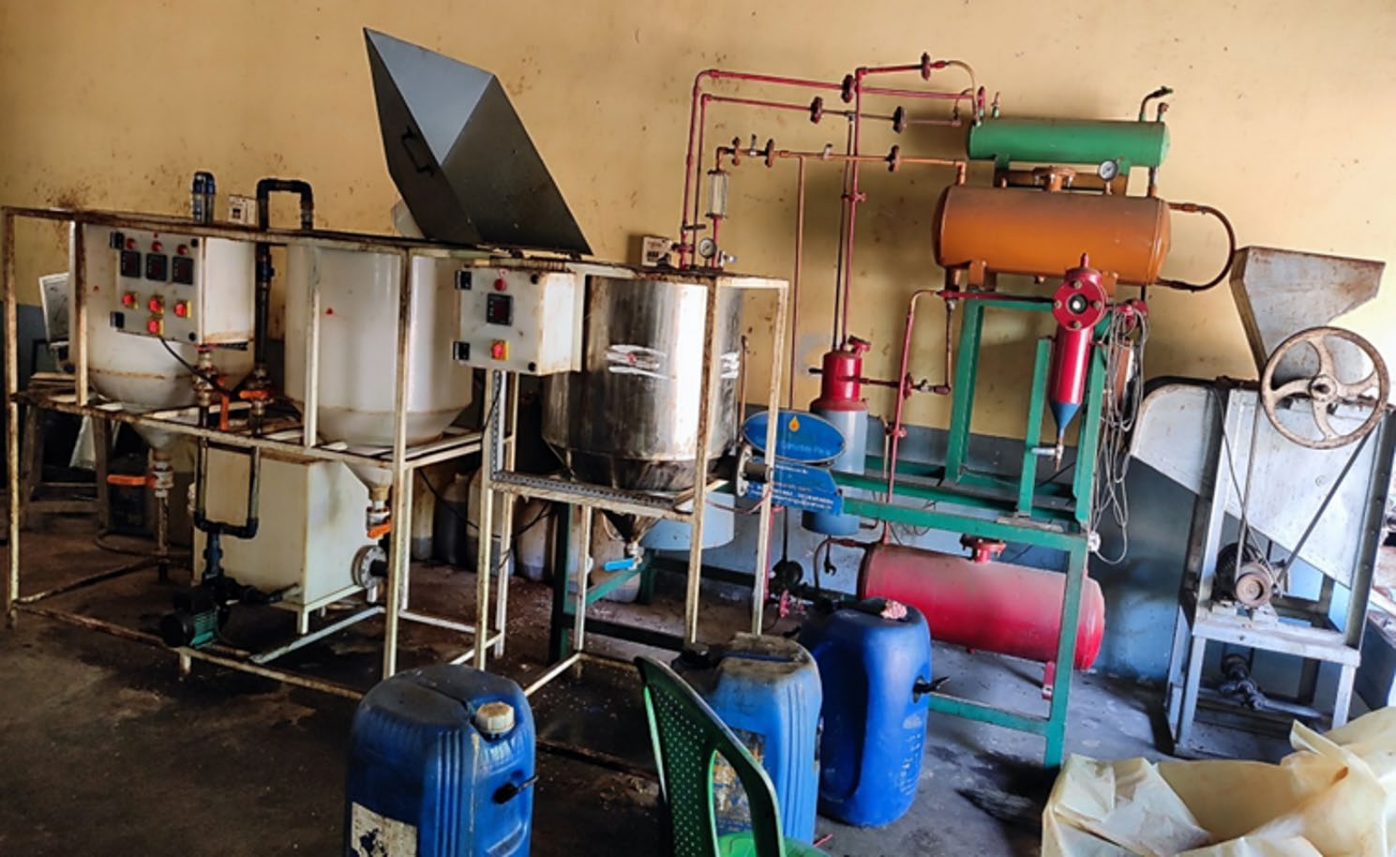
Experimental setup for the transesterification process to convert UTO into biodiesel

**Fig. 2:**
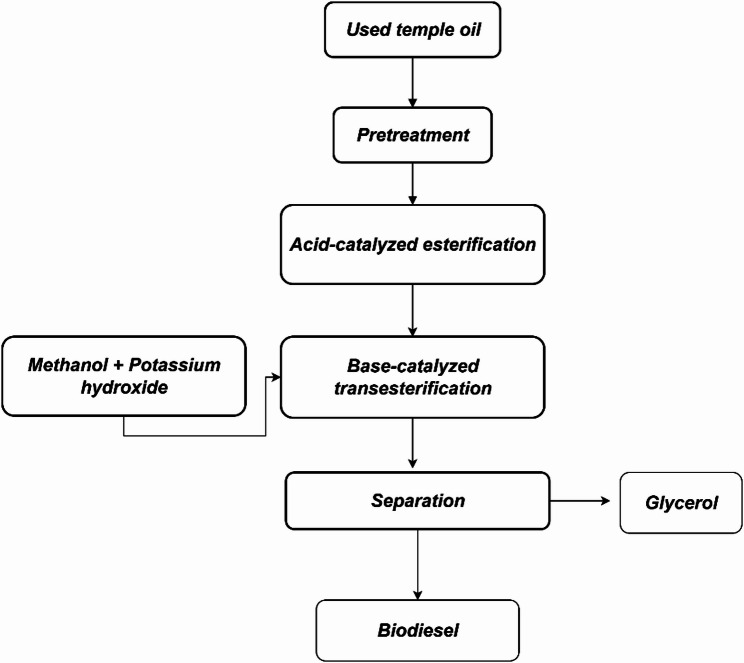
Flowchart showing the stepwise transesterification process for converting UTO into biodiesel

The preparation of biodiesel from UTO involves a transesterification process [76], as illustrated in Fig. 1. Samples of UTO were collected from several major temples and subjected to an initial filtration step to remove food residues and other impurities. Analysis of the oil indicated a free fatty acid (FFA) content of 3.8%, which is above the 2% threshold for direct, single-step, base-catalyzed transesterification. To address this limitation, the process began with an acid-catalyzed esterification step [77] designed to reduce the FFA content to below 1%. Methanol and sulfuric acid were employed as catalysts in this stage. Once the FFA level was reduced, the oil was further processed using methanol and potassium hydroxide using a base-catalyzed transesterification step [78]. This stage converted the triglycerides into methyl esters, producing glycerol as a byproduct, where the flowchart presented in Fig. 2. Overall, the transesterification process effectively decreased the viscosity of the oil [79], improving its suitability for application in diesel engines without the need for modifications.

### Fuels used

In this study, five different fuel blends were selected to evaluate engine performance. The first was standard diesel, used as a reference fuel. The second blend, B20UTOME, consisted of 20% used temple oil methyl ester (UTOME) and 80% diesel. To further enhance combustion efficiency and reduce emissions, B20UTOME was modified by incorporating CeO₂ nano additives at three different concentrations: 50 ppm, 75 ppm, and 100 ppm. These nano-additive-enriched blends, designated as B20UTOME50CeO₂, B20UTOME75CeO₂, and B20UTOME100CeO₂, were prepared to examine the catalytic influence of CeO₂ on improving fuel efficiency and emission characteristics, thereby offering more sustainable alternatives to conventional diesel.

### Preparation of fuel blends

**Fig. 3:**
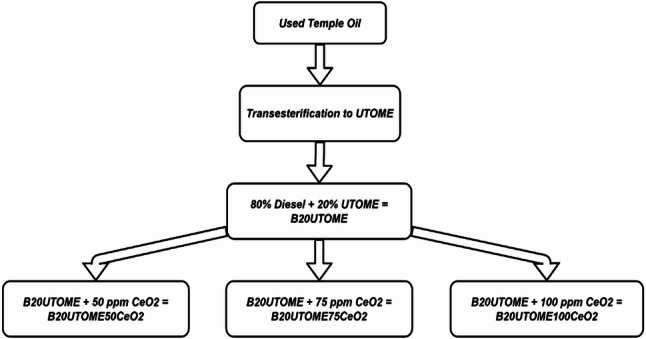
Schematic representation of the preparation of B20 biodiesel blends from UTO with varying concentrations of CeO₂ nanoparticles

In this study, UTO was repurposed through a transesterification process to produce UTOME, which was then blended with conventional diesel in an 80:20 ratio to form B20UTOME (see Fig. 3). To further enhance the fuel properties and reduce emissions, CeO₂ nanoparticles were added to the B20UTOME blend in varying concentrations of 50 ppm, 75 ppm, and 100 ppm, resulting in B20UTOME50CeO₂, B20UTOME75CeO₂, and B20UTOME100CeO₂, respectively. These nano-additive-enhanced blends were prepared to assess the catalytic effect of CeO₂ on combustion efficiency and emission reductions, leveraging the improved atomization and oxygen content of the nanoparticles. The different concentrations were selected to investigate the influence of CeO₂ on emissions and performance metrics, providing insights into the potential of B20UTOME with CeO₂ as a cleaner alternative fuel.

### Properties of fuels: diesel, UTO, UTOME, B20UTOME, B20UTOME50CeO₂, B20UTOME75CeO₂, and B20UTOME100CeO₂

The physicochemical properties of Diesel, UTO, UTOME, and their CeO₂ nanoparticle blends are detailed in Table 1. Observations indicate that the properties of B20UTOME, specifically its density, heating value, and cetane number, closely resemble those of diesel. This alignment suggests B20UTOME’s viability as a suitable alternative fuel. It has a comparable density and kinematic viscosity, ensuring smooth flow and combustion in diesel engines. Additionally, its higher heating value and cetane number are sufficient for efficient energy output [80] and ignition quality [81], making B20UTOME a sustainable substitute for diesel. The incorporation of CeO₂ nanoparticles at concentrations of 50 ppm, 75 ppm, and 100 ppm leads to a further increase in the fuel’s cetane number and heating value, which facilitates more complete combustion. These improvements suggest that B20UTOME enhanced with nano-additives may offer improved performance and safety characteristics when compared to the base blend.

**Table 1:** Properties of fuels: Diesel, UTO, UTOME, B20UTOME, B20UTOME50CeO₂, B20UTOME75CeO₂, and B20UTOME100CeO₂

Sl. No	Property	Diesel	UTO	UTOME	B20 UTOME	B20 UTOME 50CeO₂	B20 UTOME75 CeO₂	B20 UTOME100 CeO₂
1	Density (kg/m³)	850	912	868	860	861	862	863
2	Kinematic viscosity @ 40 °C (mm²/s)	2.50	25.80	5.30	3.60	3.62	3.64	3.66
3	Iodine value (mg I₂/100 g)	–	130	90	105	105	105	105
4	Acid number (mg KOH/g)	0.30	3.60	0.75	0.55	0.54	0.54	0.53
5	Higher heating Value (MJ/kg)	43.00	38.50	39.20	40.00	40.15	40.25	40.35
6	Cetane number	46	45	50	48	48.5	49	49.5
7	Flash point (°C)	56	165	110	75	77	78	80

### Properties of nano additives

**Fig. 4:**
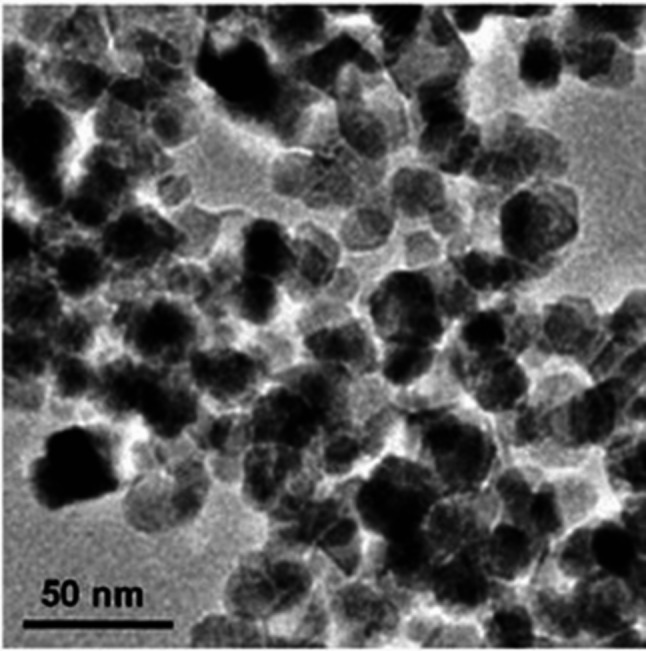
TEM image of CeO₂ nano additives

The physicochemical properties of nano-additives, specifically CeO₂ and Al₂O₃, are delineated in Table 2. CeO_2_ has a pale-yellow colour [82], with a spherical or cubic morphology [83], and an average particle size of 20 nm [84]. It has a high oxygen storage capacity (500 µmol/g) [85] and significant catalytic activity [86], making it highly effective in enhancing combustion [87] and reducing emissions. Additionally, CeO_2_ has a thermal conductivity of around 12 W/mK, suitable for applications in fuel additives [88]. Ultrasonication was employed to ensure homogeneous dispersion of CeO₂ nanoparticles within the fuel blends [89]. The preparation involved an initial 10-minute magnetic stirring phase, followed by 20 min of ultrasonication to achieve a stable and consistent distribution of the nanoparticles in the biodiesel blends. Figure 4 shows a TEM image of CeO₂ nano additives, which have a melting point of 2,400 °C, a density of 6.5 g/cm³, and an APA of 20–30 nm.

**Table 2:** Properties of nano additives: CeO_2_ and Al_2_O_3_

Property	Al_2_O_3_	CeO_2_
Colour	White	Yellowish or Pale Yellow
Morphology	Spherical or Irregular	Spherical or Cubic
Average particle size	30 nm	20 nm
Specific surface area	100 m²/g	80 m²/g
Oxygen storage capacity	5 µmol/g	500 µmol/g
Thermal conductivity	~ 30 W/m·K	~ 12 W/m·K
Catalytic activity	Low (5 µmol/g)	High (500 µmol/g)

### Experimental procedure

**Fig. 5:**
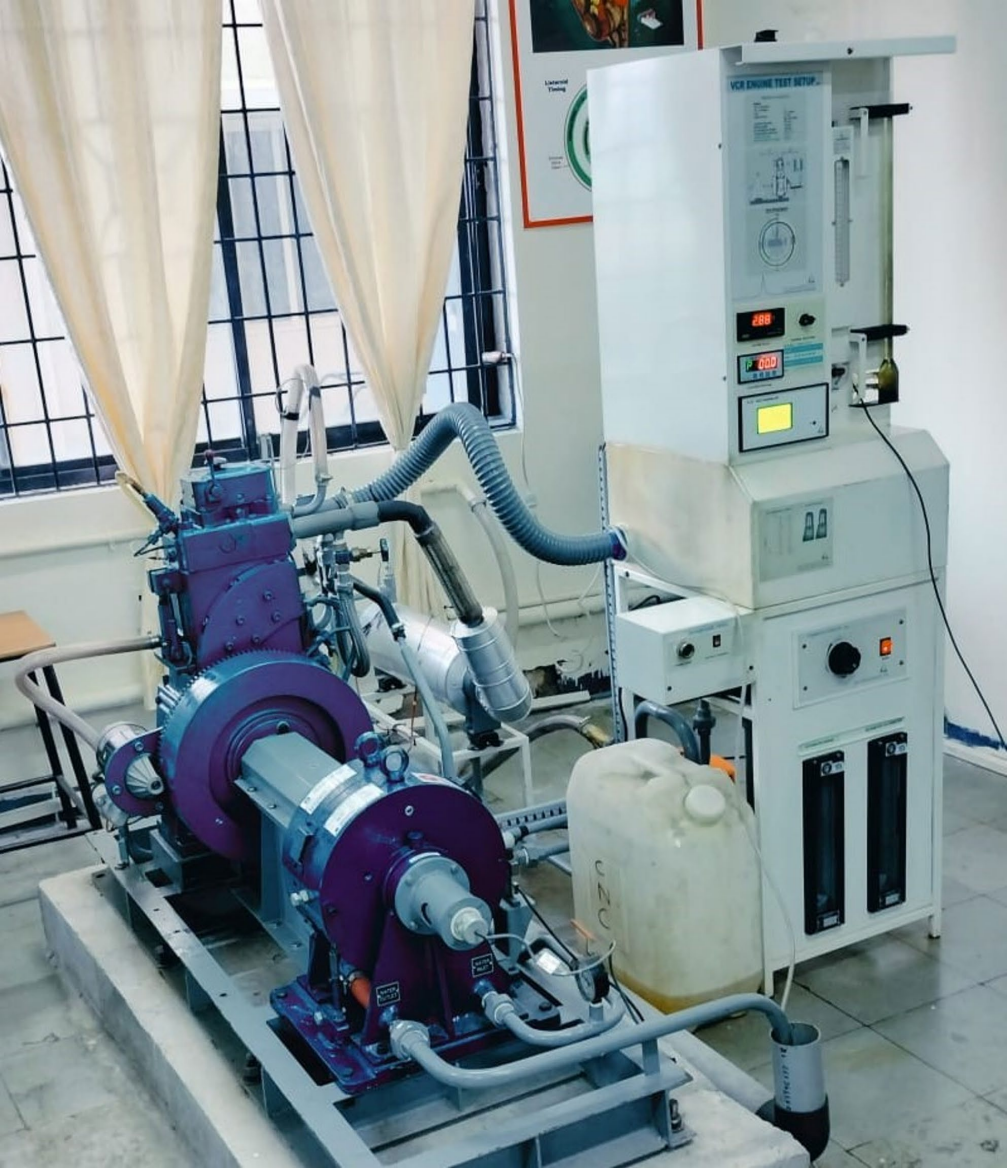
Experimental setup

**Fig. 6:**
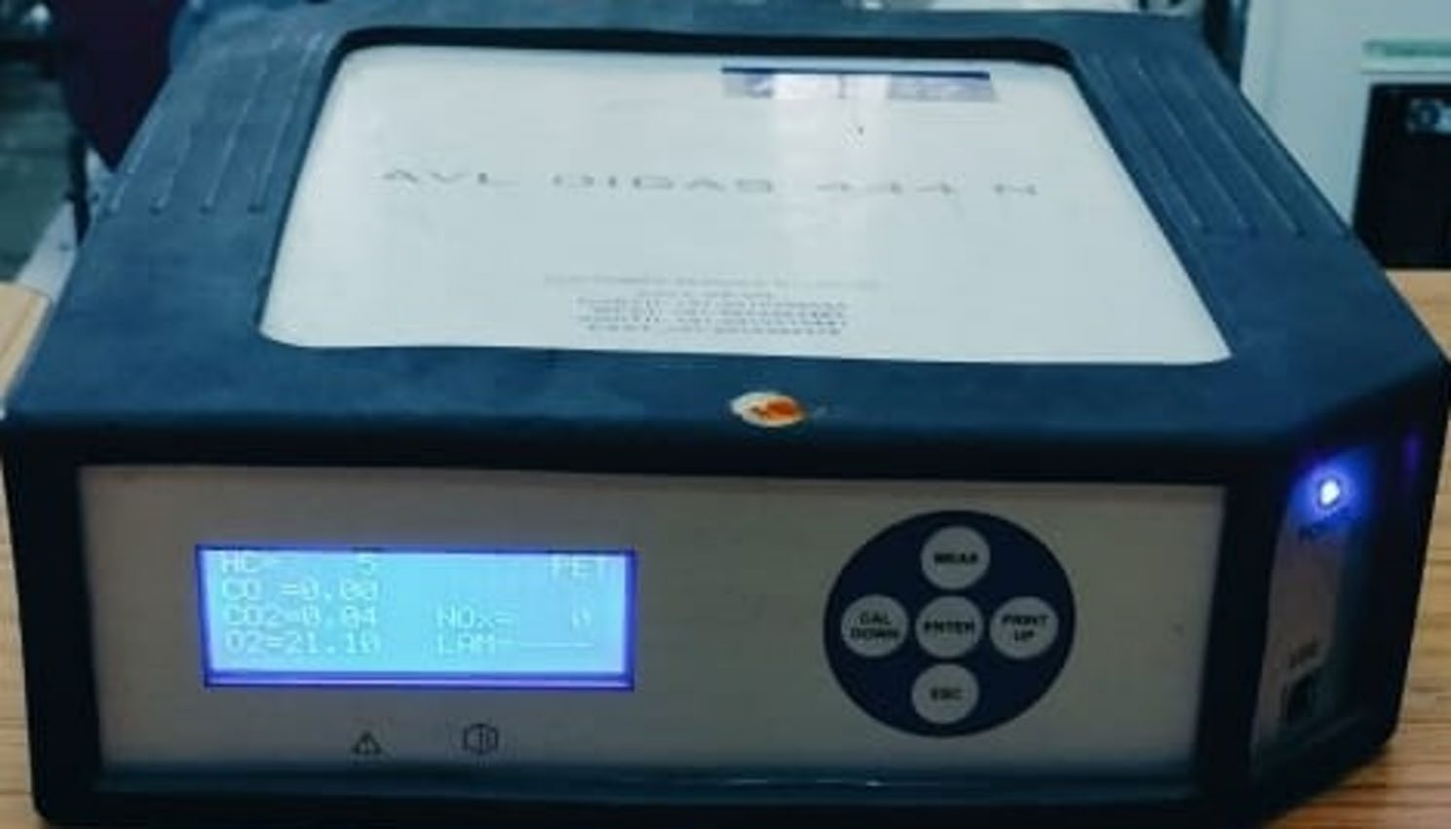
AVL Digas 444 N gas analyzer

The present experimental setup is shown in Fig. 5. A computerized test setup, incorporating a variable compression ratio diesel engine, was used to acquire measurements by maintaining a constant engine speed and adjusting the applied load. Data were collected over three trials for subsequent analysis and interpretation. The specifications of computerized VCR diesel engine test rig are mentioned in Table 3. The AVL DIGAS 444 N (Fig. 6) is a versatile gas analyzer used for emission testing, engine development, and environmental monitoring. It measures key exhaust gases such as CO, CO_2_, HC, O_2_, and NO_x_ .

**Table 3:** Specifications of the computerized VCR diesel engine test rig

Specification	Details
Engine type	1 cylinder, 4 stroke, constant speed, water-cooled, diesel engine
Power	3.5 kW @ 1500 rpm
Compression ratio	12–18:1
Cylinder bore	87.50 mm
Stroke length	110.00 mm
Connecting rod length	234.00 mm
Swept volume	661.45 cc
Orifice diameter	20.00 mm
Orifice coefficient of discharge	0.6
Dynamometer type	Eddy current
Dynamometer arm length	185 mm

### Data preparation and gradient boosting regressor model implementation

**Fig. 7:**
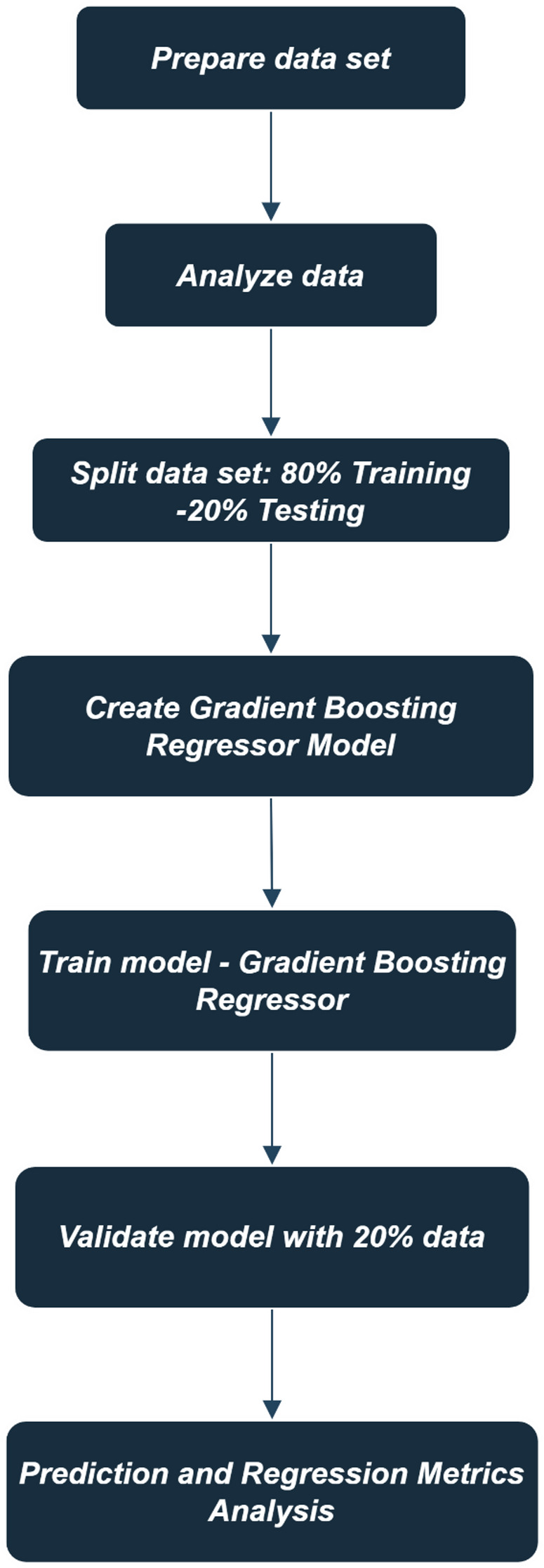
Data preparation and gradient boosting regressor model implementation

The procedure outlined above describes the steps for implementing a gradient boosting regressor model shown in Fig. 6. First, the data set is prepared and analysed to ensure it is suitable for modelling. The data is then split into training and testing sets with an 80%-20% ratio. Following this, a gradient boosting regressor model is created and trained on the 80% training data. The model’s performance is validated using the remaining 20% of the data, and the process concludes with prediction and regression metrics analysis to evaluate model accuracy and effectiveness (Fig. 7).

This research implemented a machine learning model to predict performance, combustion, and emission characteristics. A gradient boosting regressor was trained using a dataset of 25 readings from Fish oil biodiesel blends with nanoparticle additives, developed with Python libraries such as Scikit-learn, NumPy, Pandas, and Matplotlib. Input parameters included engine load, biodiesel blend ratio, nano additive concentration, oxygen storage capacity, fuel density, fuel viscosity, cetane number, calorific value, and flash point. Output variables comprised BTE, SFC, cylinder pressure (CP), net heat release (NHR) rate, and emission characteristics. The gradient boosting regressor utilized the hyperparameters: n_estimators = 100, learning_rate = 0.1, and max_depth = 3.

## Results and discussion

### Performance characteristics

#### BTE

**Fig. 8:**
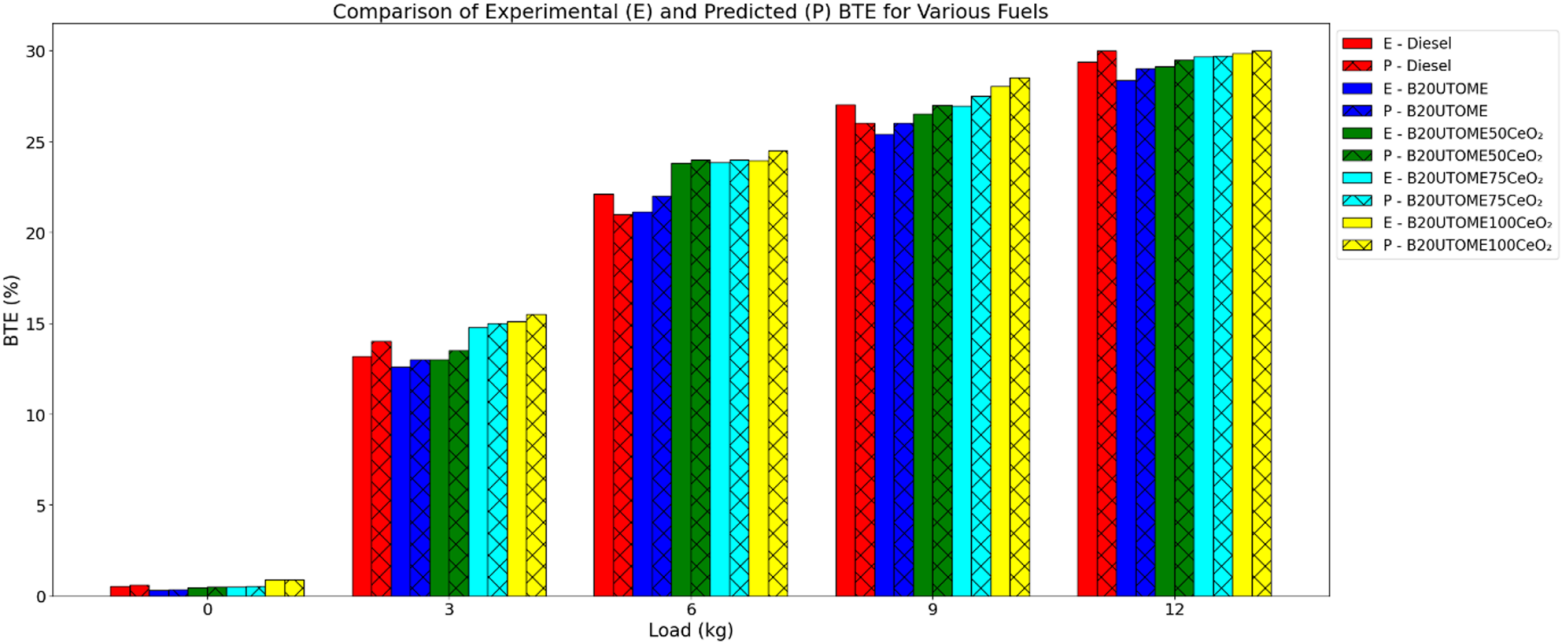
Variation of BTE with load at constant speed

Based on the experimental findings presented in Fig. 8, the addition of CeO₂ nano additives to the B20UTOME blend significantly enhances BTE, achieving a level competitive with pure diesel. The high surface area to volume ratio of these nanoparticles improves combustion by promoting better fuel atomization and faster evaporation, leading to more efficient energy conversion. The comparison of predicted and experimental BTE values, demonstrates that the predicted values closely match the experimental values. This validation, conducted using the gradient boosting regressor model, achieved a high R-squared score of 0.9842 and a low mean squared error of 1.5291, confirming the model’s reliability in estimating BTE. These findings show the effectiveness of the B20UTOME100CeO₂ blend as a superior alternative for enhancing combustion efficiency compared to both B20UTOME without additives and pure diesel.

#### SFC

**Fig. 9:**
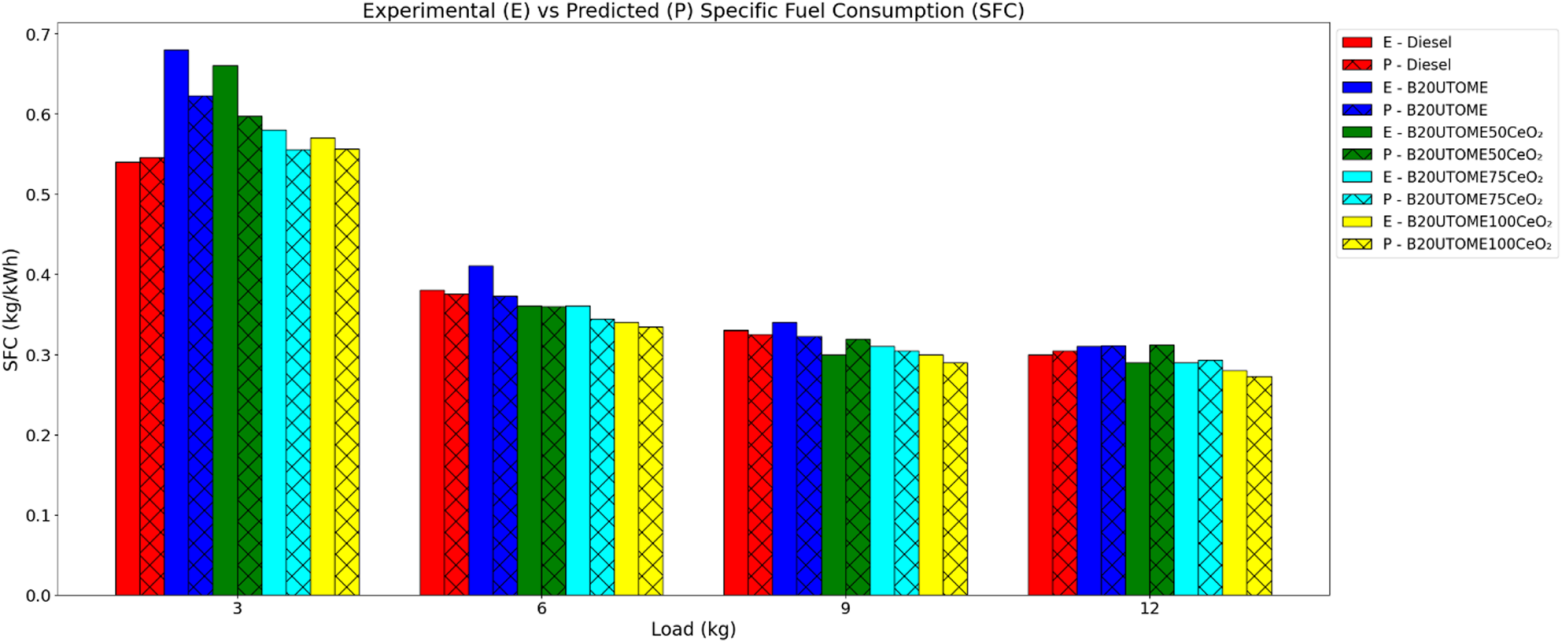
Variation of SFC with load at constant speed

The experimental results depicted in Fig. 9 demonstrate that incorporating CeO₂ nano additives into the B20UTOME blend leads to a notable reduction in SFC, suggesting an improvement in fuel utilization. The elevated surface area-to-volume ratio of the nanoparticles facilitates enhanced combustion by promoting improved fuel atomization and accelerated evaporation, which consequently lowers fuel consumption for equivalent power generation. A comparison between the predicted and experimental SFC values indicates a strong agreement between the predicted outcomes and the empirical data. This validation, employing the gradient boosting regressor model, yielded a high R-squared value and a minimal mean squared error, thus confirming the model’s precision in SFC estimation. These findings present the efficacy of the B20UTOME100CeO₂ blend in achieving enhanced fuel economy relative to both B20UTOME without additives and traditional diesel fuel.

### Combustion characteristics

#### CP

**Fig. 10:**
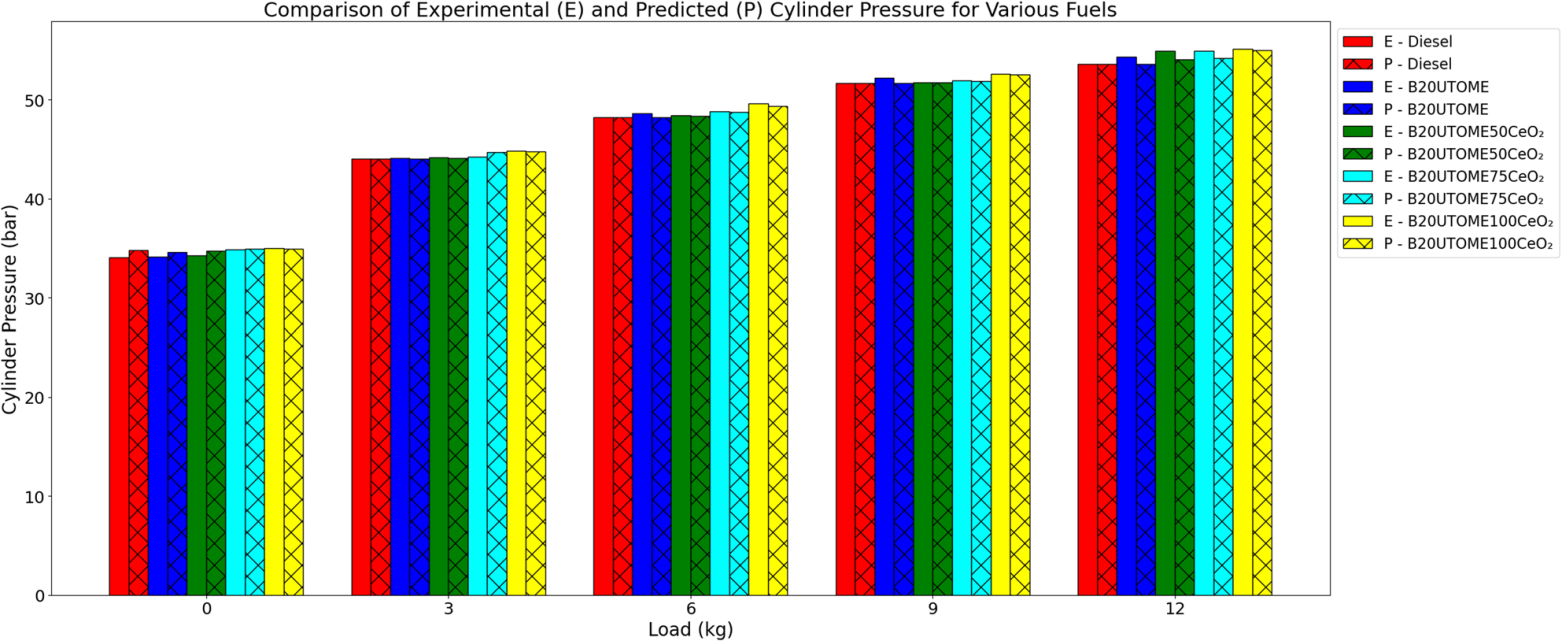
Variation of maximum CP with load at constant speed

The fluctuation of CP with load at constant speed is depicted in Fig. 10. It emphasizes that all fuels have higher CP with load because of enhanced combustion efficiency. B20UTOME blends have a slightly higher CP than pure diesel, which displays a constant increase in CP. The peak CP of B20UTOME is further increased by adding CeO₂ nano additions; for all loads, B20UTOME100CeO₂ achieves the maximum CP. CeO₂ nanoparticles, which promote fuel atomization and dispersion, are responsible for the improvement in decreased ignition delay and improved combustion. A gradient boosting regressor model with a low mean squared error (0.2136) and high R-squared score (0.9957) was used to validate the predicted CP values, which showed great prediction accuracy and near match experimental results.

#### NHR

**Fig. 11:**
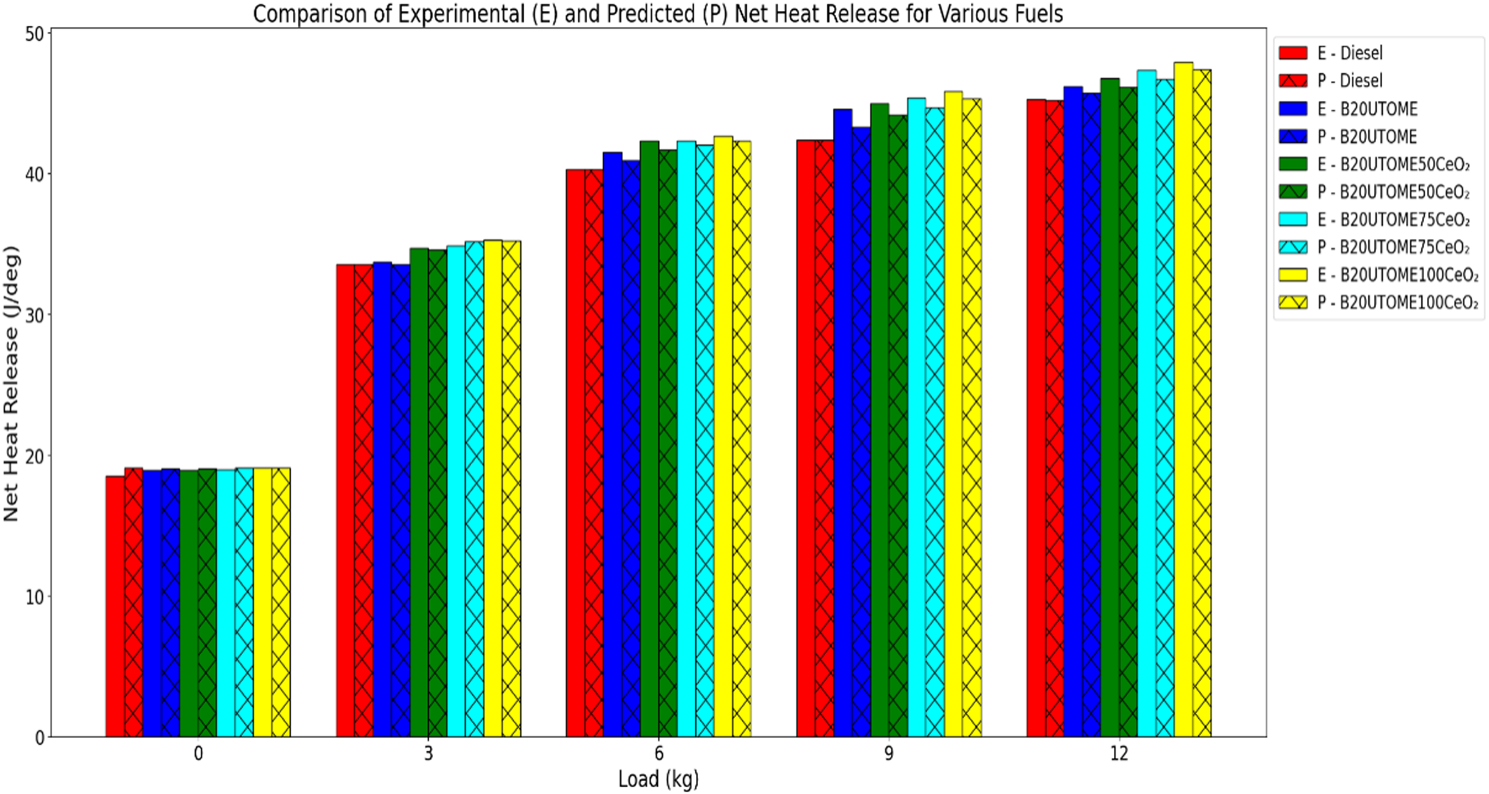
Variation of maximum NHR with load at constant speed

NHR values compared to diesel at similar loads presented in Fig. 11. The addition of CeO₂ nano additives to B20UTOME further enhances the NHR, with the B20UTOME100CeO₂ blend achieving the highest NHR across all loads, demonstrating the most substantial improvement. The increase in NHR with CeO₂ additives can be attributed to the enhanced catalytic activity provided by the nanoparticles, which promote better fuel atomization, reduce ignition delay, and lead to a more complete and efficient combustion process. As a result, B20UTOME blends with CeO₂ additives exhibit higher NHR compared to both pure diesel and B20UTOME without additives, highlighting the beneficial impact of CeO₂ on combustion efficiency and heat release. The comparison of predicted and experimental NHR values indicates that the predicted values closely align with the experimental data. The gradient boosting regressor model used for validation achieved a very low mean squared error of 0.0998 and an exceptionally high R-squared score of 0.9990, indicating remarkable predictive accuracy and reliability.

### Emission characteristics

#### CO

**Fig. 12:**
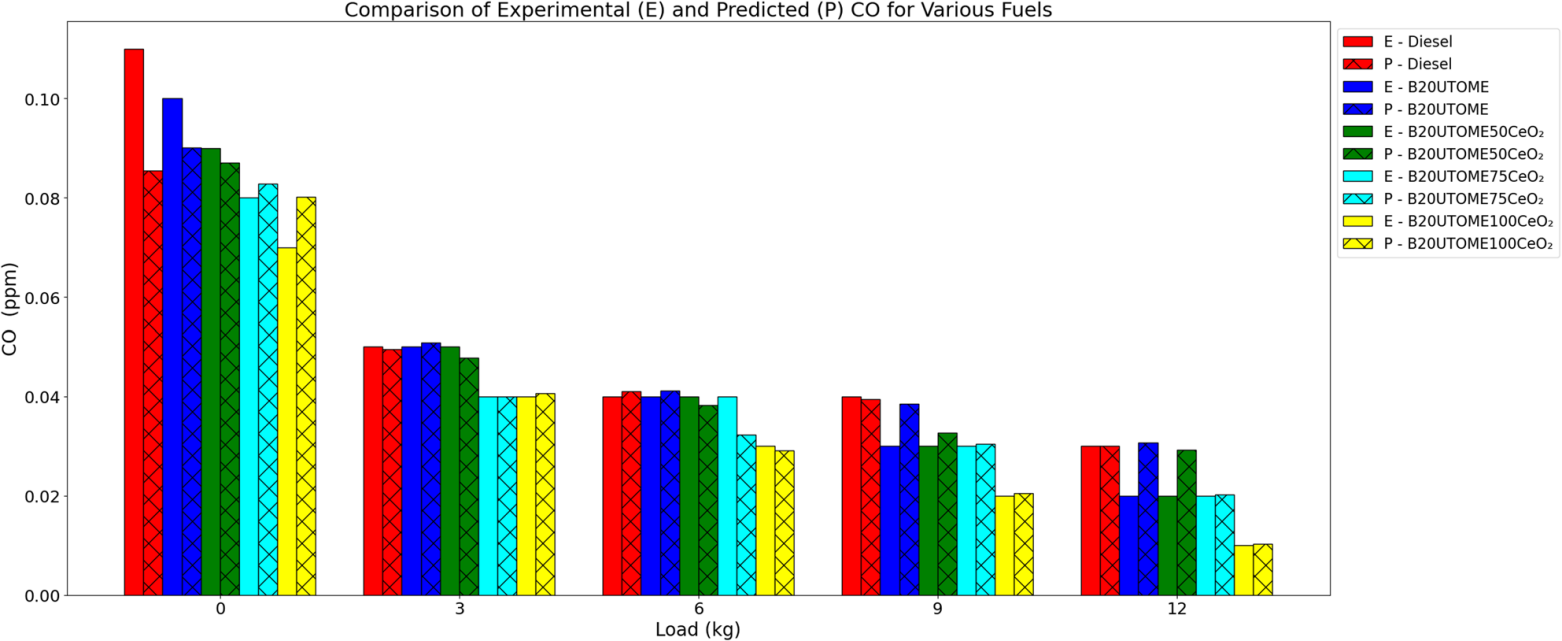
Variation of CO with load at constant speed

The relationship between load and CO emissions at constant speed is depicted in Fig. 12. For all fuels, CO emissions decrease as load increases, suggesting increased combustion efficiency. B20UTOME blends emit less CO than pure diesel; for all loads, B20UTOME100CeO₂ has the lowest emissions. This decrease is brought about by the oxygen in CeO₂ nanoparticles, which promotes air-fuel mixing for more thorough burning, increases combustion, and shortens ignition delay. A gradient boosting regressor model with a low mean squared error (0.0002) and R-squared score (0.7881) showed that the predicted CO levels closely matched the experimental data, indicating dependable predictive performance and emphasizing the contribution of CeO₂ to cleaner combustion. Recognizing CO as a toxic pollutant contributing to hypoxia [90] and significant health risks [91], the reduction of its emissions holds considerable environmental [92] and health importance [93]. Therefore, the diminished CO emissions resulting from B20UTOME100CeO₂ not only enhance engine operational efficiency [94], but also alleviate detrimental biomedical consequences for human health [95].

####  HC

**Fig. 13:**
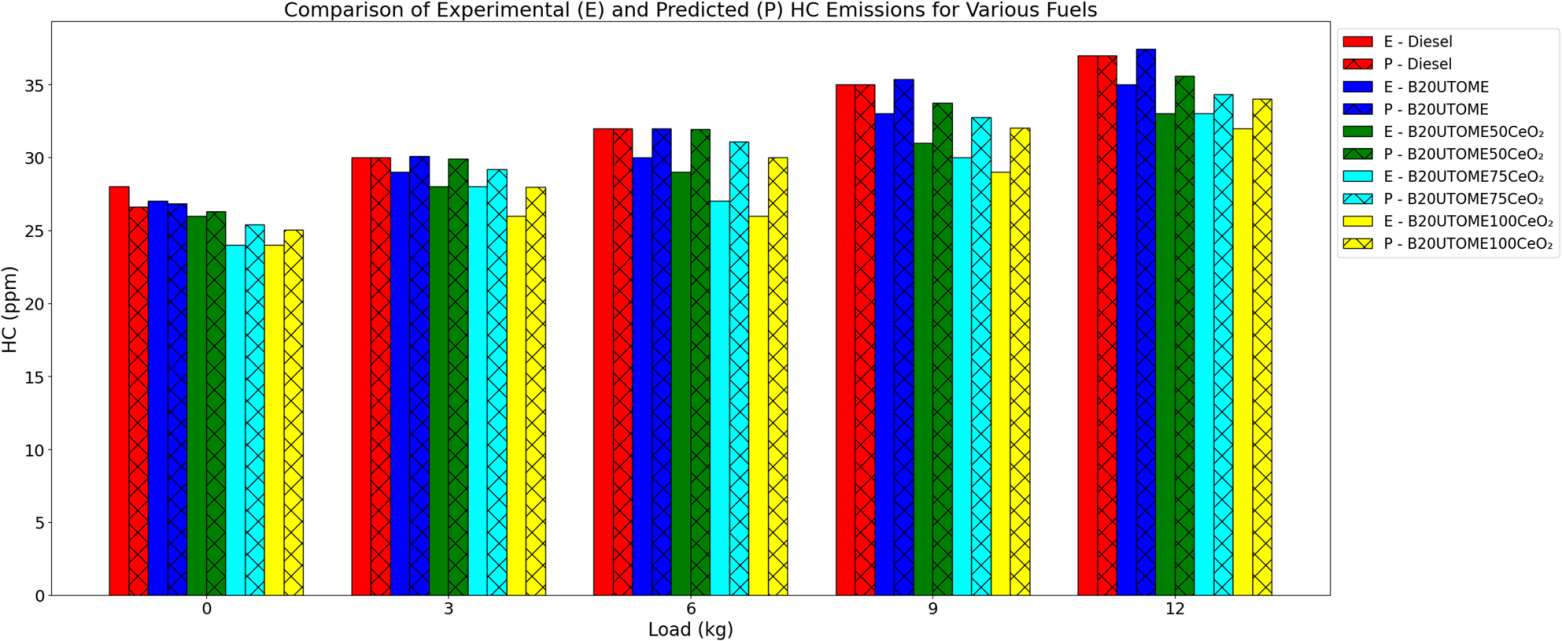
Variation of HC with load at constant speed

HC emissions vary with load at constant speed, as shown in Fig. 13. While B20UTOME blends show slightly lower emissions than pure diesel at every load level, HC emissions rise with load for all fuels. When CeO₂ nano additions are added to B20UTOME, HC emissions are greatly decreased; the reductions are greater at higher CeO₂ concentrations. Because the nanoparticles promote more complete combustion, better fuel atomization, and increased combustion efficiency, the B20UTOME100CeO₂ blend exhibits the lowest HC emissions across all loads. A gradient boosting regressor model with a mean squared error of 0.7647 and an R-squared score of 0.9247 validates the predicted HC emissions, which show great predictive accuracy and emphasize the catalytic function of CeO₂ in lowering emissions. Unburned HC represent significant environmental hazards [96], contributing to respiratory ailments [97], neurological impairments [98], and potential long-term carcinogenic impacts [99]. Consequently, the observed decrease in HC emissions attributable to B20UTOME100CeO₂ signifies not only enhanced combustion efficiency [100] but also a mitigation of severe biomedical risks associated with extended exposure to these compounds [101].

#### NO_x_

**Fig. 14:**
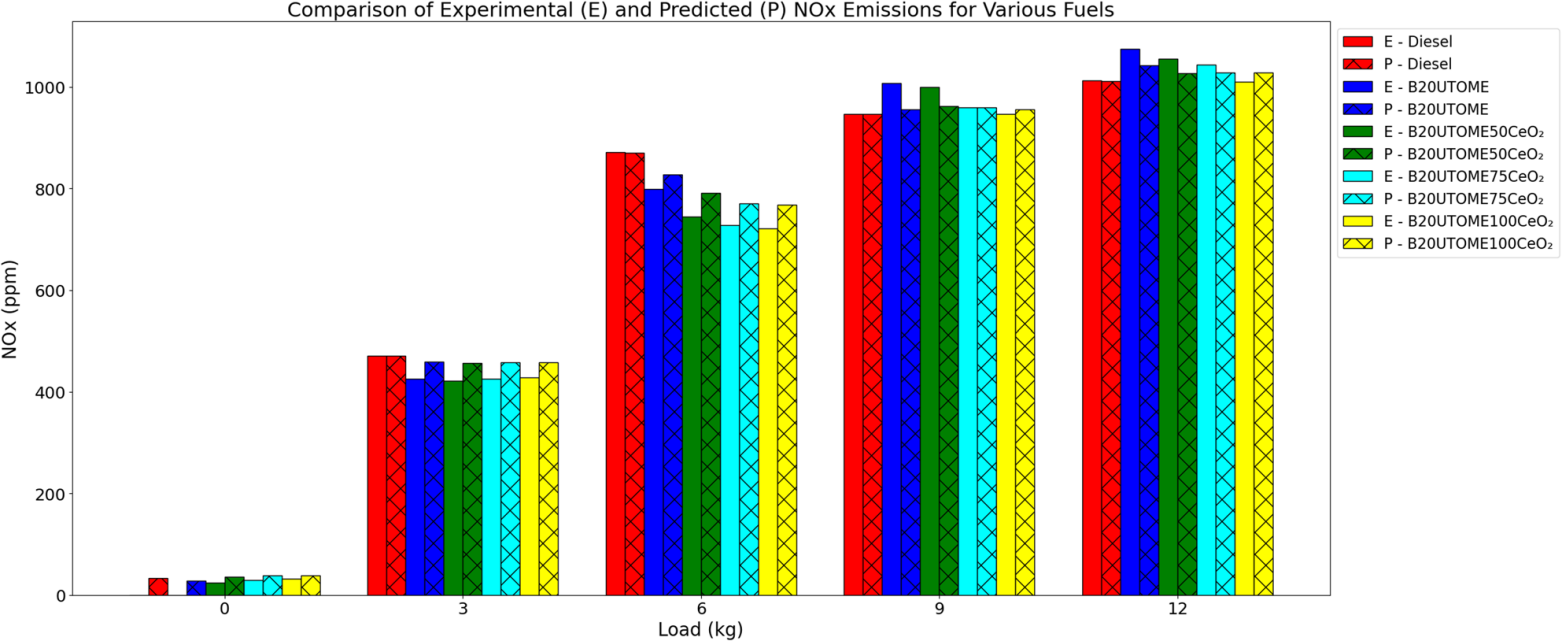
Variation of NO_x_ with load at constant speed

The fluctuation of NO_x_ emissions with load at constant speed is shown in Fig. 14. For all fuels, NO_x_ emissions increase with load because of increased combustion pressures and temperatures. Pure diesel exhibits a consistent increase in NO_x_ emissions, however B20UTOME blends generate higher NO_x_ emissions at higher loads. This is because biodiesel’s oxygen content improves combustion. Higher quantities of CeO₂ result in larger reductions in NO_x_ emissions when added to B20UTOME as CeO₂ nano additives. Because CeO₂ enhances combustion efficiency and thermal management, lowering peak temperatures and NO_x_ production, the B20UTOME100CeO₂ blend generates the lowest NO_x_ emissions of all the blends. Predicted values closely match experimental data, validated by a gradient boosting regressor model with an R-squared score of 0.9882, confirming strong predictive accuracy. Increased concentrations of NO_x_ are established to induce damage to lung tissue [102], compromise respiratory functions [103], and elevate the probability of developing cardiovascular diseases [104]. The documented decrease in NO_x_ resulting from the nanoparticle-enhanced fuel shows its biomedical relevance in alleviating respiratory health risks [105] and facilitating cleaner combustion [106].

### Limitations of the present study

While the findings of this research highlight the potential of B20UTOME biodiesel blends enhanced with CeO₂ nanoparticles, several limitations must be acknowledged. First, the study was conducted using a single-cylinder, constant-speed, variable compression ratio diesel engine under laboratory conditions. Although this setup provides controlled and repeatable results, it may not fully replicate the performance and emission behavior of multi-cylinder, variable-speed engines commonly used in real-world applications. Consequently, the scalability of the results to commercial diesel engines remains to be validated [107].

Second, the experimental dataset was relatively limited in size, comprising 25 readings, and was supplemented with machine learning validation using a gradient boosting regressor model. While the model demonstrated strong predictive accuracy with high R² values and low mean squared errors, the robustness of the predictions could be further enhanced by expanding the dataset to include broader operating conditions such as transient load variations [108], cold-start behavior [109], and long-duration engine trials [110]. The reliance on a relatively small dataset introduces the possibility of overfitting and constrains the generalizability of the machine learning outcomes [111].

Third, the investigation focused primarily on CeO₂ as the nano-additive, without exploring the synergistic effects of combining multiple nanoparticles, such as Al₂O₃, TiO₂, or GO derivatives. Emerging evidence suggests that hybrid nano-additives may offer superior catalytic [112], thermal [113], and oxidative properties [114], potentially leading to even greater improvements in performance [115] and emission reduction [116]. The exclusion of such comparisons limits the scope of the study to CeO₂ alone.

Fourth, although the study incorporated biomedical perspectives by linking reduced emissions of CO, HC, and NO_x_ to public health benefits, no direct toxicological [117] or epidemiological assessments [118] were carried out. The biomedical implications were therefore inferred from existing literature rather than measured through exposure-based experiments [119] or in vitro [120]/in vivo [121] studies. This reliance on secondary data constrains the ability to establish direct causal links between emission reductions and health outcomes.

Fifth, although the study employed regression metrics for validating machine learning predictions, no formal statistical hypothesis testing such as analysis of variance (ANOVA) [92] was carried out to quantify the significance of the observed differences across fuel blends. ANOVA is particularly valuable in determining whether improvements in parameters such as brake thermal efficiency, specific fuel consumption, or emissions are statistically significant or fall within experimental uncertainty. The absence of ANOVA-based validation limits the ability to generalize the findings with statistical confidence and may raise concerns about experimental reproducibility. Future work incorporating ANOVA and post-hoc tests would therefore be beneficial in establishing the robustness of the reported improvements.

Finally, the economic and lifecycle aspects of CeO₂-enhanced biodiesel production were not assessed. Parameters such as the cost of nanoparticle synthesis [122], energy input during ultrasonication [123], long-term fuel stability [124], and the environmental footprint of nanoparticle production [125] were beyond the scope of this study but remain critical for evaluating the feasibility of large-scale adoption. A comprehensive techno-economic and life-cycle analysis would therefore be necessary to complement the promising technical and biomedical results presented here [126].

Taken together, these limitations do not diminish the value of the present findings but rather highlight avenues for further research. Future investigations should involve large-scale engine trials under variable operating conditions, expanded datasets for machine learning validation, incorporation of ANOVA for statistical confirmation of experimental results, exploration of hybrid nano-additives, direct biomedical toxicity testing, and integrated economic and environmental assessments. Addressing these gaps will provide a more holistic understanding of the potential of CeO₂-enhanced biodiesel as a sustainable and health-conscious alternative to fossil-derived diesel fuels.

## Conclusion

This research study explores the potential of B20 biodiesel blends derived from UTOME enhanced with CeO₂ nanoparticle additives as a sustainable alternative to conventional diesel fuel. This is the first study to combine B20UTOME with 100 ppm CeO₂ and validate results using gradient boosting regressor model. The B20UTOME100CeO₂ blend exhibited significant enhancements in BTE, a marked decrease in SFC, and improvements in combustion characteristics, particularly higher CP and NHR. Emission analysis revealed substantial reductions in HC, CO, and NO_x_, resulting from more thorough combustion processes. Considering the established negative biomedical consequences of vehicular emissions, including respiratory ailments, cardiovascular issues, and cognitive impairments, the reduction in harmful pollutants signifies positive public health outcomes from employing these cleaner fuels. A gradient boosting regressor model was trained on experimental data comprising fish oil biodiesel blends with nanoparticle additives, utilizing Python libraries. Validation of the performance, combustion, and emission parameters through high R² scores and low mean squared error confirms the reliability of these findings. The results of this study establish B20UTOME with 100ppm CeO₂ additives as a viable and efficient alternative to traditional diesel, offering improved efficiency, cleaner combustion, and reduced environmental impact.

## Data Availability

All data generated or analyzed during this study are included in this published article. No additional datasets were generated or analyzed beyond the contents of the manuscript. No generated any crystallographic or macromolecular structure data in this study.

## Abbreviations

ANOVA: Analysis of variance
BTE: Brake thermal efficiency
CNT: Carbon nanotube
CP: Cylinder pressure
FFA: Free fatty acid
NER: Net heat release
PAH: Polycyclic aromatic hydrocarbons
PM_2.5_: Fine particulate matter
SFC: Specific fuel consumption
UTO: Used temple oil
UTOME: Used temple oil methyl ester

## Chemical Symbols

Al₂O₃: Aluminium oxide
CeO₂: Cerium oxide
CO: Carbon monoxide
CO_2_: Carbon dioxide
GO: Graphene oxide
HC: Hydrocarbons
NO_x_: Nitrogen oxides
NO_2_: Nitrogen dioxide
TiO₂: Titanium dioxide

## Declarations

### Declarations of AI use

The authors declare the use of generative artificial intelligence (AI) and AI-assisted technologies in the preparation of this manuscript. Specifically, ChatGPT (version GPT-4, OpenAI) was used to improve the readability and language of the article. The AI tool was not used for data analysis, interpretation of results, or drawing scientific conclusions. All content has been reviewed and verified by the authors to ensure accuracy and integrity.
